# ﻿A new species of *Andricus* Hartig, 1840 (Hymenoptera, Cynipidae) from China, with references to DNA taxonomy and *Wolbachia* infection

**DOI:** 10.3897/zookeys.1134.89267

**Published:** 2022-12-07

**Authors:** Yin Pang, Cheng-Yuan Su, Jun-Qiao Zhu, Xiao-Hui Yang, Jia-Lian Zhong, Dao-Hong Zhu, Zhiwei Liu

**Affiliations:** 1 Laboratory of Insect Behavior and Evolutionary Ecology, College of Life Science and Technology, Central South University of Forestry and Technology, Changsha, Hunan, 410004, China Central South University of Forestry and Technology Changsha China; 2 School of Life Sciences, Hunan Normal University, Changsha, Hunan, 410081, China Hunan Normal University Changsha China; 3 Biological Sciences Department, Eastern Illinois University, Charleston, Illinois 61920, USA Eastern Illinois University Charleston United States of America

**Keywords:** *Andricuselodeoides*, gall wasp, phylogeny, *Quercusserrata*, taxonomy

## Abstract

In the present paper, a new species of cynipid gall wasp, *Andricuselodeoides* Liu & Pang, is described from several provinces in southern China. The new species is closely related to the recently redescribed *A.mairei* (Kieffer, 1906). In addition to differences in adult and gall morphology, the new species is also readily separated by COI sequences, with a 6.2–8.9% genetic distance between populations of the new species and those of *A.mairei*. A contrasting difference in sex ratios was also observed between the two species, with *A.elodeoides* extremely female-biased (95.5–97.8% female) while *A.mairei* male-biased to more balanced (5.4–43.5% female). PCR screening for *Wolbachia* infection further revealed contrasting infection rates between populations of *A.elodeoides* and *A.mairei*: the *Wolbachia* infection rate was 0% in *A.elodeoides* and 100% in *A.mairei*. Cytoplasmic incompatibility induced by *Wolbachia* is proposed as a potential mechanism of speciation of the sympatric *A.elodeoides* and *A.mairei*.

## ﻿Introduction

The genus *Andricus* Hartig, 1840 (Hymenoptera, Cynipoidea, Cynipidae, Cynipini) is the largest genus of the oak-gall wasp tribe Cynipini, currently comprising approximately 400 known species (Melika 2006) and making up 40% of the known species diversity of the tribe (Wachi et al. 2011). The genus is predominantly Holarctic, with the highest recorded species diversity from the Nearctic and Western Palearctic (Wang et al. 2013). However, a number of new species of the genus have also been described in the last decade or so from Mesoamerica in the Neotropical realm (Melika et al. 2009a, b; Pujade-Villar et al. 2016) and the Oriental realm (Tang et al. 2009, 2012; Wang et al. 2013; Pujade-Villar et al. 2014; Ide et al. 2018). In Eastern Asia, which stretches from the Palearctic to the Oriental, 19 *Andricus* species are known (Ide et al. 2018; Penzes et al. 2018; Pujade-Villar et al. 2020).

The unusually high diversity of *Andricus* species among all the genera of the tribe Cynipini may be an artifact, as the genus is not well defined and often has been treated as a “trash can” genus in Cynipini (Melika 2006). In their taxonomic review of the world genera of cynipine wasps, Melika and Abrahamson (2002) treated several previously recognized genera as junior synonyms of the genus because of the lack of reliable diagnostic characteristics, rather than because of the existence of defining synapomorphies (Melika and Abrahamson 2002). One of the synonymized genera, *Druon* Kinsey, 1937 has since been re-established as a valid genus (Cuesta-Porta et al. 2022). Although multiple phylogenetic studies involving Cynipini have invariably shown *Andricus* to be paraphyletic or polyphyletic (Stone and Cook 1998; Cook et al. 2002; Rokas et al. 2003; Stone and Schönrogge 2003; Liljeblad et al. 2008; Ronquist et al. 2015), the current concept of the genus is still largely based on that of Melika and Abrahamson (2002).

One of the genera synonymized with *Andricus* Hartig, 1840 by Melika and Abrahamson (2002) is *Parandricus* Kieffer, 1906, which is known from China and includes a single species, *P.mairei* Kieffer, 1906. A detailed redescription of the species was done based on specimens collected from Zhejiang Province of China because the original type of *P.mairei* Kieffer, 1906 was lost and the original description was inadequate by today’s standards (Pujade-Villar et al. 2020). In the last few years, we have reared a large series of specimens that apparently belong to multiple, known or unknown, species of *Andricus*, including *A.mairei* (Kieffer, 1906) (Yang et al. 2012). In the present paper, we describe a new species from that series of *Andricus* specimens and provide a detailed comparison between it and the apparently closely related *A.mairei* (Kieffer 1906). We also sequenced the mitochondrial COI gene for both species for DNA barcoding as well as the nuclear 28S D2 region to place the new species within the current phylogenetic framework of all *Andricus* species that had both COI and 28S sequences available.

## ﻿Materials and methods

### ﻿Specimen collection

The galls of gall wasps were collected from 12 locations in six provinces in southern China in late spring to early summer from 2012 to 2019 (Table 1). The collected galls were cage-reared at room temperature in the laboratory of the College of Life Science and Technology, Central South University of Forestry and Technology (CSUFT) and checked daily for emergence. Adult wasps were directly preserved in 100% ethanol within 2 days after emergence and stored in freezer at −80 °C until being retrieved for morphological and molecular studies.

**Table 1.** Collection information, female ratio and *Wolbachia* infection in *A.elodeoides* sp. nov. and *A.mairei*.

Location(code)	Coordinates	Date of gall collection	Date of adult emergence	Insect species	Female: male	*Wolbachia* infect frequency (%)
Xinyang, Henan (XY)	32°02'N, 113°53'E	May, 2012	May, 2012	*A.mairei*	8: 46 (14.8%*)	100 (20)^†^
				*A.elodeoides*	64: 2 (97.0%)	0 (20)
Jinzhai, Anhui (JZ)	31°38'N, 115°58'E	May, 2014	May, 2014	*A.mairei*	64: 318 (16.8%)	100 (20)
				*A.elodeoides*	224: 5 (97.8%)	0 (40)
		May, 2015	May, 2015	*A.mairei*	12: 63 (16.0%)	100 (20)
				*A.elodeoides*	78: 2 (97.5%)	0 (20)
		May, 2016	May, 2016	*A.mairei*	19: 213 (8.2%)	100 (20)
				*A.elodeoides*	86: 3 (96.6%)	0 (20)
		May, 2017	May, 2017	*A.mairei*	9: 43 (17.3%)	100 (20)
				*A.elodeoides*	123: 4 (96.9%)	0 (20)
		May, 2018	May, 2018	*A.mairei*	29: 512 (5.4%)	–
				*A.elodeoides*	128: 6 (95.5%)	–
		May, 2019	May, 2019	*A.mairei*	46: 612 (7.0%)	–
				*A.elodeoides*	224: 8 (96.6%)	–
Shucheng, Anhui (SHC)	31°21'N, 116°04'E	May, 2016	May, 2016	*A.mairei*	34: 104 (24.6%)	100 (20)
				*A.elodeoides*	426: 13 (97.0%)	0 (40)
		May, 2017	May, 2017	*A.mairei*	6: 46 (11.5%)	100 (20)
				*A.elodeoides*	91: 2 (97.8%)	0 (20)
		May, 2018	May, 2018	*A.mairei*	16: 65 (19.8%)	100 (20)
				*A.elodeoides*	73: 3 (96.1%)	0 (20)
		May, 2019	May, 2019	*A.mairei*	9: 56 (13.8%)	100 (20)
				*A.elodeoides*	129: 6 (95.6%)	0 (20)
Taihu, Anhui (TH)	30°34'N, 116°04'E	May, 2016	May, 2016	*A.mairei*	12: 32 (27.3%)	100 (20)
				*A.elodeoides*	94: 3 (96.9%)	0 (40)
Wuhan, Hubei (WH)	30°31'N, 114°31'E	May, 2014	May, 2014	*A.mairei*	8: 12 (40.0%)	100 (20)
				*A.elodeoides*	166: 6 (96.5%)	0 (40)
Changsha, Hunan CS)	28°25'N, 113°07'E	May, 2016	May, 2016	*A.mairei*	102: 136 (42.9%)	100 (20)
		May, 2017	May, 2017	*A.mairei*	258: 349 (42.9%)	–
		May, 2018	May, 2018	*A.mairei*	121: 157 (43.5%)	–
Suichang, Zhejiang (SUC)	28°37'N, 119°19'E	April, 2018	May, 2018	*A.elodeoides*	79: 2 (97.5%)	0 (30)
				*A.mairei*	124: 987 (11.2%)	100 (20)
Qingyuan, Zhejiang (QY)	27°44'N, 119°15'E	April, 2018	May, 2018	*A.elodeoides*	76: 3 (96.2%)	0 (20)
				*A.mairei*	23: 245 (8.6%)	100 (20)
Zhenghe, Fujian (ZH)	27°23'N, 118°2'E	April, 2018	May, 2018	*A.mairei*	66: 568 (10.4%)	100 (20)
Zhouning, Fujian (ZN)	27°13'N, 119°20'E	April, 2018	May, 2018	*A.mairei*	13: 86 (13.1%)	100 (20)
Guiding, Guizhou (GD)	26°37'N, 107°14'E	May, 2017	Jun, 2017	*A.mairei*	6: 24 (20.0%)	100 (20)
Shaoguan, Guangdong (SG)	24°59'N, 113°01'E	April, 2017	May, 2017	*A.mairei*	34: 256 (11.7%)	100 (20)

* Percentage of females; ^†^ The number in parentheses refers to the number of insect individuals screened.

### ﻿Morphological observations

Specimens for conventional morphological examination were air dried at room temperature before mounting. Specimens mounted to pinned triangle-card paper were studied under a stereomicroscope (SZX7, Olympus, Japan) and automatically stacked photographs were taken with Leica M205C microscope system (Leica, Germany) equipped with Leica DMC6200 digital camera connected to a computer. Additional specimens were dissected out and transferred to diluted ammonia (5%) and kept overnight to remove debris that might interfere with observation. Cleansed parts were then rinsed in distilled water and dehydrated gradually through 25%, 50%, 75%, and 100% ethanol solutions, and finally stored in 100% ethanol. Dehydrated specimen parts were air-dried before being mounted onto aluminum stub (Ted Pella, Redding, CA, USA) with copper conductive tape (3M). Gold-coated specimens were examined with JEOL JSM-6380Lv SEM (JEOL, Japan) at CSUFT with 15 KV voltage, and selected frames were saved as digitized high-resolution TIFF images.

We follow Ronquist and Nordlander (1989) and Ronquist (1995) for structural terminology, Melika (2006) for measurement definitions, and Harris (1979) for surface sculpture descriptions. Abbreviations: F1 and F2 = the first and second flagellomeres, respectively; POL (post-ocellar distance) = the distance between the inner margins of the posterior ocelli; OOL (ocellar-ocular distance) = the distance from the outer margin of a posterior ocellus to the inner margin of the compound eye; LOL (lateral-frontal ocelli distance) = the distance between anterior and lateral ocelli. Type specimens are deposited in Insect Collection, Central South University of Forestry and Technology (CSUFT), Changsha, Hunan.

### ﻿DNA extraction and sequencing

Three individuals from each population of two gall wasp species were used for DNA extraction. The insects were washed in sterile water before DNA extraction to avoid surface contamination. Total DNA was extracted from each individual using SDS/proteinase K digestion and a phenol-chloroform extraction. Extracted DNA pellets were air dried, resuspended in 50 µl sterile water, and then stored at 4 °C before being processed for PCR and sequencing.

For phylogenetic analysis, we chose a specific region of the mitochondrial cytochrome c oxidase subunit I gene (COI) and the nuclear large ribosomal subunit gene (28S), which were amplified with the primes HCO-2198 (5′-TAAACTTCAGGGTGACCAAAAAATCA-3′) and LCO-1490 (5′-GGTCAACAAATCATAAAGATATTGG-3′) (Folmer et al. 1994), and D2F (5′-CGTGTTGCTTGATAGTGCAGC-3′) and D2R (5′ TCAAGACGGGTCCTGAAAGT 3′) (Dowton and Austin 2001), respectively. This gene fragment was chosen because of its suitability for recovering inter- and intrageneric phylogenies within the Hymenoptera in general and Cynipidae in particular (Rokas et al. 2002) as well as sequence availability for a reasonable number of congeneric species from public depositories. The PCR mixture was composed of 1 µl of PrimeSTAR HS DNA Polymerase (Takara Biomedical Technology Co., Dalian, China), 10 µl of buffer, 4 µl of dNTPs, 1 µl of each primer, and 2 µl of DNA with water added to achieve a total volume of 50 µl. The amplification was conducted using a C1000 Touch thermal cycler (Bio-Rad, Hercules, CA, United States). The cycling conditions were 98 °C for 3 min, 35 cycles of 98 °C for 10 s, 50–57 °C for 30 s, and 72 °C for 1 min. Amplified PCR products were sequenced in both directions using an ABI 3730XLDNA sequencer (Applied Biosystems, Foster City, CA, USA) with M13F/R at Wuhan Icongene Co., Ltd. The sequences have been deposited in GenBank under the following accession numbers: COION803612 to ON803631 and 28S ON911591 to ON911610 (Table 2).

**Table 2.** Sequences of mitochondrial COI and nuclear 28S genes used in the phylogenetic analysis.

Gall wasp	COI	28S D2	Reference
*Andricuscaputmedusae*	DQ012619	EF030040	Liljeblad (2002)
*Andricuscurvator*	DQ012621	AF395155	Liljeblad (2002)
*Andricuscoriarius*	DQ012620	DQ012579	Liljeblad (2002)
*Andricuscrystallinus*	MT179597	MT183614	Pujade-Villar et al. (2020)
*Andricushakonensis*	MT179612	MT183628	Pujade-Villar et al. (2020)
*Andricuskollari*	AF395176	AF395156	Rokas et al. (2002)
*Andricuspictus*	DQ012625	DQ012583	Liljeblad (2002)
*Andricusquercusstrobilana*	DQ012617	DQ012576	Liljeblad (2002)
*Andricusrochai*	MT179600	MT183671	Pujade-Villar et al. (2020)
*Andricusxishuangbannaus*	MT179618	MT183634	Pujade-Villar et al. (2020)
*Andricusmairei* (ILV92)	MT179620		Pujade-Villar et al. (2020)
(ILV90)	MT179616		
(ILV87)	MT179614		
(ILV86)	MT179613		
(ILV32)	MT179604		
(ILV31)	MT179603		
(ILV30)	MT179602		
(ILV91)	MT179617		
*Andricusmairei*	ON803612–ON803624	ON911591–ON911603	Present study
*Andricuselodeoides*	ON803625–ON803631	ON911604–ON911610	Present study
*Melikaiellabicolor*	MT179619	MT183623	Pujade-Villar et al. (2020)
*Dryocosmusliui*	MG754067	MN633412	Pang et al. (2018); Pang et al. (2020)

### ﻿Phylogenetic analysis

The COI and 28S gene sequences of 11 species of *Andricus* (including eight populations of *A.mairei*) and *Dryocosmusliui* and *Melikaiellabicolor* (as outgroups) were retrieved from GenBank (https://www.ncbi.nlm.nih.gov/genbank/) (Table 2). The final dataset consists of 14 species including the new species and outgroup. Multiple sequence alignment was performed using ClustalW (Thompson et al. 1994) implemented in MEGA 11.0 (Kumar et al. 2016) using default parameters. ClustalW aligned sequences were then visually edited in MEGA 11.0 and trimmed, resulting a final aligned length of 1154 bp nucleotides for COI and 1053 bp nucleotides for 28S.

The final dataset was subjected to MEGA 11.0 for evaluation of best fit nucleotide substitution model (Nei and Kumar 2000) using the maximum likelihood (ML) method with default settings except that we used “very strong” branch swap filter. Phylogenetic analysis was conducted using MrBayes 3.2.6 x64 for Windows (Ronquist et al. 2012) (Bayesian inference method, BI), assuming a generalized Time-reversible (GTR) model with gamma distributed rate variation across sites (+G) based on best fit nucleotide substitution model evaluation performed earlier. For Bayesian analysis, two independent runs were performed with the default priors and MCMC parameters except the following: nst = 6, rates = gamma, MCMC runs comprised 10 million generations sampled at every 1,000 generations with 30% burn-in time. Convergence was achieved as being diagnosed by the average standard deviation of split frequencies between the two independent runs (<0.01) and PSRF values (1 with < 1% deviation). The final tree from both analyses was rooted with *D.liui* and *M.bicolor* based on published phylogeny of Cynipidae (Ronquist et al. 2015).

To compare directly with a recent study on *A.mairei* and related species based solely on COI (Pujade-Villar et al. 2020), we also performed a phylogenetic analysis based on COI only to include the sequences of *A.mairei* from various populations published in that study.

Finally, the pair-wise genetic distance in the COI sequences from all populations of *A.elodeoides* and *A.mairei*, and other two *Andricus* species were calculated, using the MEGA 11.0 (Kumar et al. 2016).

### ﻿*Wolbachia* screening

*Wolbachia* infections were screened by PCR with the *Wolbachia*-specific primers wsp-81F and wsp-691R that amplify a 575–625 bp fragment of the *wsp* gene encoding *Wolbachia* surface protein (Zhou et al. 1998). To verify the presence of *Wolbachia* infection in *A.elodeoides*, *gatB*, *coax*, *ftsZ*, and *hcpA* genes were amplified for various populations using the respective primers reported by Baldo et al. (2006). Amplification methods and conditions were as previously described (Hou et al. 2020).

## ﻿Results

### ﻿Taxonomy

#### ﻿ Andricus elodeoides

Taxon classificationAnimaliaHymenopteraCynipidae

Liu & Pang sp. nov.

208DF5CE-4BF7-5411-ADFA-A3D37BC736CE

https://zoobank.org/8FD547C-C534-4F23-8FE8-1E60987D8959

Figs 1–6 , 7–13

##### Type materials.

***Holotype*** ♀; ***Paratypes***: 10♀, 8♂♂. China, Hunan Province, Changsha City (113°07'N, 28°25'E), 2011-V-11–20, leg. Xiao-Hui Yang, deposited in Insect Collection, Central South University of Forestry and Technology (CSUFT), Changsha, Hunan.

##### Etymology.

The species epithet derived from *Elodea*, the genus name of the aquatic plants well known as waterweeds, referring to the superficial resemblance of the cluster of galls of the species to these plants.

##### Additional materials examined.

Same data as holotype, 3♂, 3♀ (Cheng-Yuan Su leg.). Jinzhai (31°38'N, 115°58'E), Anhui province. 3♂, 3♀ (Cheng-Yuan Su leg.). Wuhan (30°31'N, 114°31'E), Hubei province. 3♂, 3♀ (Cheng-Yuan Su leg.). Suichang (28°37'N, 119°19'E), Zhejiang province. 1♂, 1♀ (Cheng-Yuan Su leg.). Xinyang (32°02'N, 113°53'E), Henan province,. 3♂, 3♀ (Cheng-Yuan Su leg.). Taihu (30°34'N, 116°04'E), Anhui province. 3♂, 3♀ (Cheng-Yuan Su leg.), Qingyuan (27°44'N, 119°15'E), Zhejiang province. 3♂, 3♀ (Cheng-Yuan Su leg.), Zhenghe (27°23'N, 118°52'E), Fujian province. 3♂, 3♀ (Cheng-Yuan Su leg.), Zhouning (27°13'N, 119°20'E), Fujian province. 3♂, 3♀ (Cheng-Yuan Su leg.), Guiding (26°37'N, 107°14'E), Guizhou province. 3♂, 3♀ (Cheng-Yuan Su leg.), Shaoguan (24°59'N, 113°01'E), Guangdong province.

##### Diagnosis.

The new species is similar to *A.mairei* (Kieffer 1906), but differs from the latter in having: 1) vertex and frons glabrate with long setae evenly-spaced on vertex and scatted on frons in the new species (Fig. 3), whereas vertex coriaceous and vertex and frons with sparse short setae in *A.mairei* (Pujade-Villar et al. 2020: fig. 1b, d); 2) male antenna F1 strongly curved medially in the new species (Fig. 10), but straight in *A.mairei* (Pujade-Villar et al. 2020: fig. 2b); 3) mesopleuron glabrous in the new species (Fig. 6), whereas with weak longitudinal striation medially in *A.mairei* (Pujade-Villar et al. 2020: fig. 3c, d, but compare with fig. 3e); 4) mature galls of *A.elodeoides* are straight and cylindrical, fully covered with dense resinous white hairs (Fig. 14), whereas the galls of *A.mairei* are curved or strongly tapering in distal half, mostly shining smooth with an apical cluster of white hairs (Fig. 15).

**Figures 1–6.**
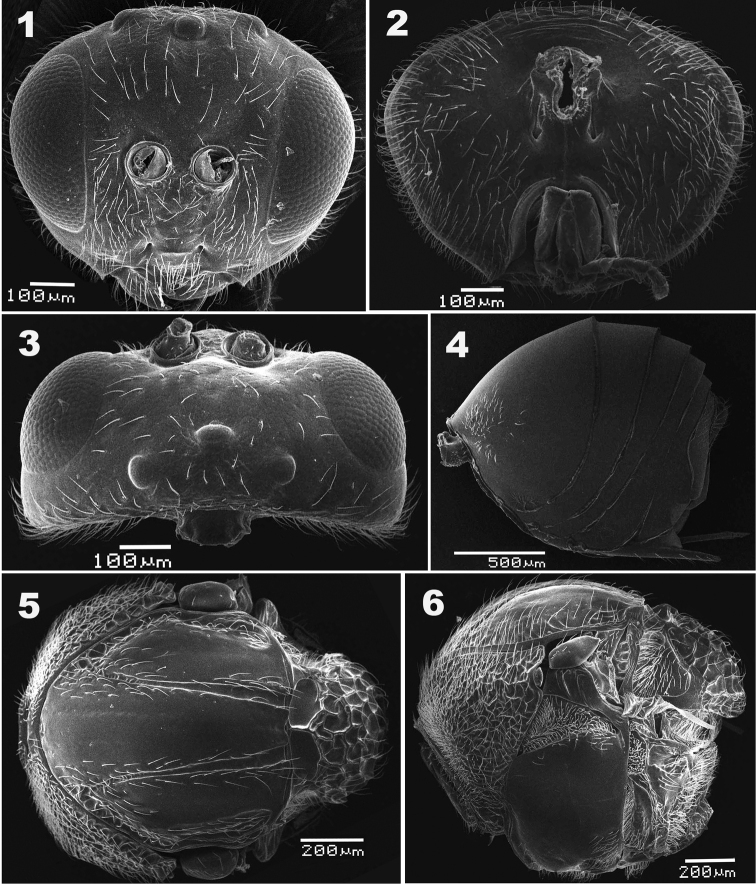
*Andricuselodeoides* sp. nov., female **1** head in anterior view **2** head in posterior view **3** head in dorsal view **4** metasoma in lateral view **5** mesosoma in dorsal view **6** mesosoma in lateral view.

##### Description.

**Female**: body length 2.6–2.8 mm (*N* = 5).

***Coloration*.** Head area of compound eyes and frons black and gena yellow. Antenna uniformly dark brown to black, except for scape, pedicel and F1 brownish yellow. Mandible, maxillar and labial palpi dark brown. Legs uniformly brownish yellow. Mesosoma black; metasoma mostly reddish brown and posteriorly black. Hypopygial spine reddish brown.

***Forewing*** with distinct veins R+Sc, R1+Sc, R1, Rs, Rs+M (somewhat faint basally), M, 2r, M+Cu1, Cu1, Cu1b and Cu1a; areolet distinct and small; marginal cell about 2.6–3.0 times as long as wide; all visible veins yellow except for the distal half of R+Sc, R1+Sc, 2r, and M. The distal half of M+Cu1 black (Fig. 12).

***Head*** coriaceous, 1.2 times as wide as high in anterior view, nearly oval, broader than mesosoma in front view and 2.2 times as broad as long in dorsal view. Gena not broadened behind eyes in dorsal view. Height of eye about 3.4 times the length of malar space. Frons glabrate with evenly spaced long setae, with ocellar triangle indistinctly rugose; lower face and malar space glabrate and distinctly setose. Clypeus distinct and impressed; epistomal sulcus distinct; anterior tentorial pits small, but distinct; clypeo-pleurostomal line distinct. Transfacial distance slightly bigger than height of eye; distance between inner margin of eye and outer rim of antennal torulus slightly wider than distance between antennal toruli, but as wide as diameter of torulus (Fig. 1). Posterior ocelli widely separated from each other, ratios of POL/OOL, POL/LOL, and LOL/OOL 2.1, 2.7 and 0.9, respectively. In dorsal view, posterior margin of anterior ocellus nearly aligned with anterior margin of posterior ocelli (Fig. 3). Vertex glabrate, covered with scattered long setae. Gena coriarious, posteriorly with sparce long setae; postgena mostly glabrate with dense setae in outer edge. Occiput very finely imbricate and setose except medially; posterior tentorial pits distinct. Gular sulci absent; area around occipital foramen glabrous (Fig. 2).

***Antenna*** filiform with 11 flagellomeres, slightly tapering toward apex; pedicel sub-spherical; relative lengths of scape, pedicel and F1-F11: 10:6:11:9:9:8:8:8:7:7:6:6:13; placoid sensillae distinctly visible on F2–F11 (Fig. 9).

**Figures 7–13.**
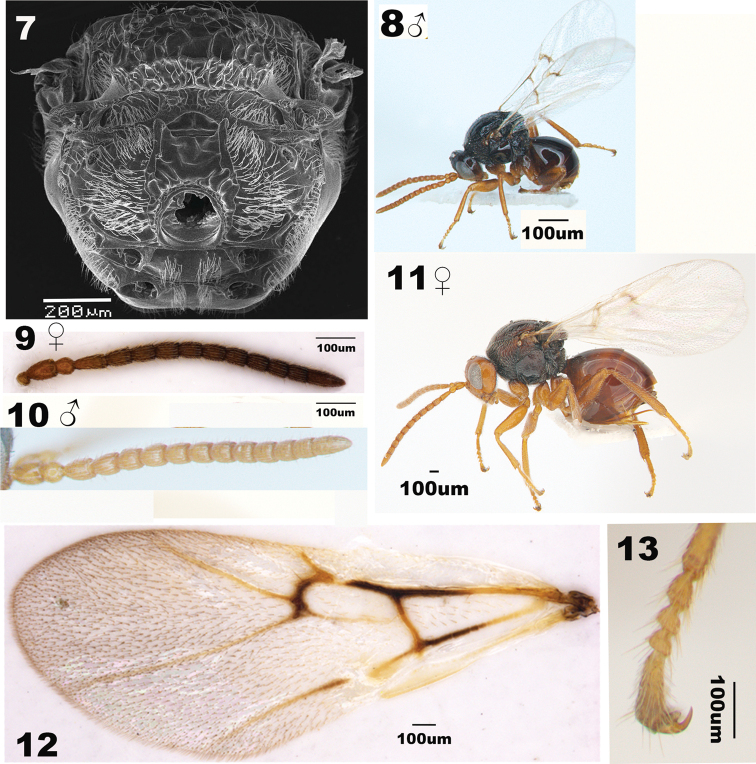
*Andricuselodeoides* sp. nov. **7** propodeum of female in dorsal view **8** general habitus of male **9** female antenna **10** male antenna **11** general habitus of female **12** female forewing **13** the claw of hind leg of female.

***Mesosoma*** longer than high in lateral view. Pronotum median length two ninth of length of outer lateral margin. Anterior plate of pronotum areolate to rugose and densely setose laterally (Fig. 6); Mesoscutum nearly as long as width measured at anterior tip of tegulae, with some small foveae and setae along outer edge. Notauli distinct and glabrous, lined with setae along sides, and slightly broadened posteriorly. Mesoscutellum broader than long, areolate-rugose and sparsely setose. Scutellar foveae deeply impressed and glabrous, separated by a median carina. Mesopleural triangle glabrate and densely setose. Metapleural sulcus reaching mesopleuron in upper 2/3 of its height; metapleuron glabrate with sparse setae (Fig. 6). Median dorsellum area rugose with dense setae. Propodeum with long and dense setae; lateral propodeal carinae distinct and parallel; median propodeal area confused-rugulose, lateral propodeal area with dense long and appressed setae (Fig. 7). Nucha short, width as long in height and lateral view, and longitudinally costate with posterior punctate-areolate ring (Fig. 6).

***Metasoma*** 1.2 times as long as high in lateral view; abdominal tergite II 1.5 times as high as long in lateral view, laterally with anterior patch of short setae; tergite VII dorsally and VIII with long setae. Prominent part of hypopygium slender, distally not pointed; and ventrally with a row of short setae (Fig. 4).

**Male**: Similar to female, but different as below. Antenna with 12 flagellomeres, length of scape 1.25 times as long as wide; pedicel almost same as long as broad. F1 strongly curved medially. Lengths of scape, pedicel and F1–F12: 10:10:7:8:8:7:7:7:7:7:7:14. Upper face black, lower face yellow (Figs 8, 10).

##### Gall.

**Figures 14, 15.**
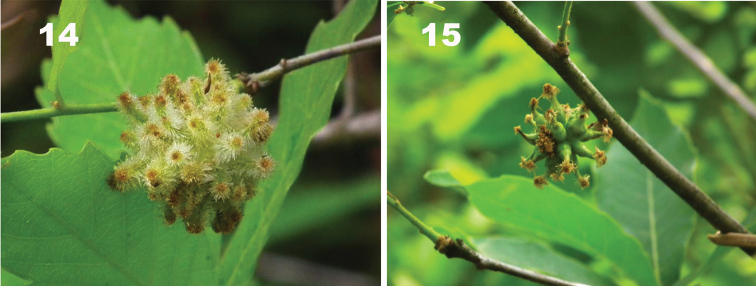
Galls on *Quercusserrata***14***Andricuselodeoides* sp. nov. **15***Andricusmairei*.

Galls are monolocular and form clusters of 50–60 galls on twigs of host plant. Galls are covered with very dense resinous white hairs, which become brown at the terminal of the galls as galls mature. Individual galls straight and cylindrical (Fig. 14), but not curved or strongly tapering in distal half as in *A.mairei* (Fig. 15).

##### Biology.

All specimens emerged from galls collected from *Quercusserrata*. The adults of the new species appeared in early to mid-May (which overlaps with the emergence period of *A.mairei*). Populations were extremely female-biased at 95.5–97.8% (while that of *A.mairei* were 5.4–43.5%) (Table 1).

##### Distribution.

The new species is currently known from China in several provinces in the middle to lower reaches of the Yangtze River, including Henan (Xinyang), Anhui (Jinzhai, Shucheng, and Taihu), Hubei (Wuhan), Hunan (Changsha and Shaoyang), and Zhejiang (Suichang and Qingyuan).

### ﻿Molecular phylogeny

**Figure 16.**
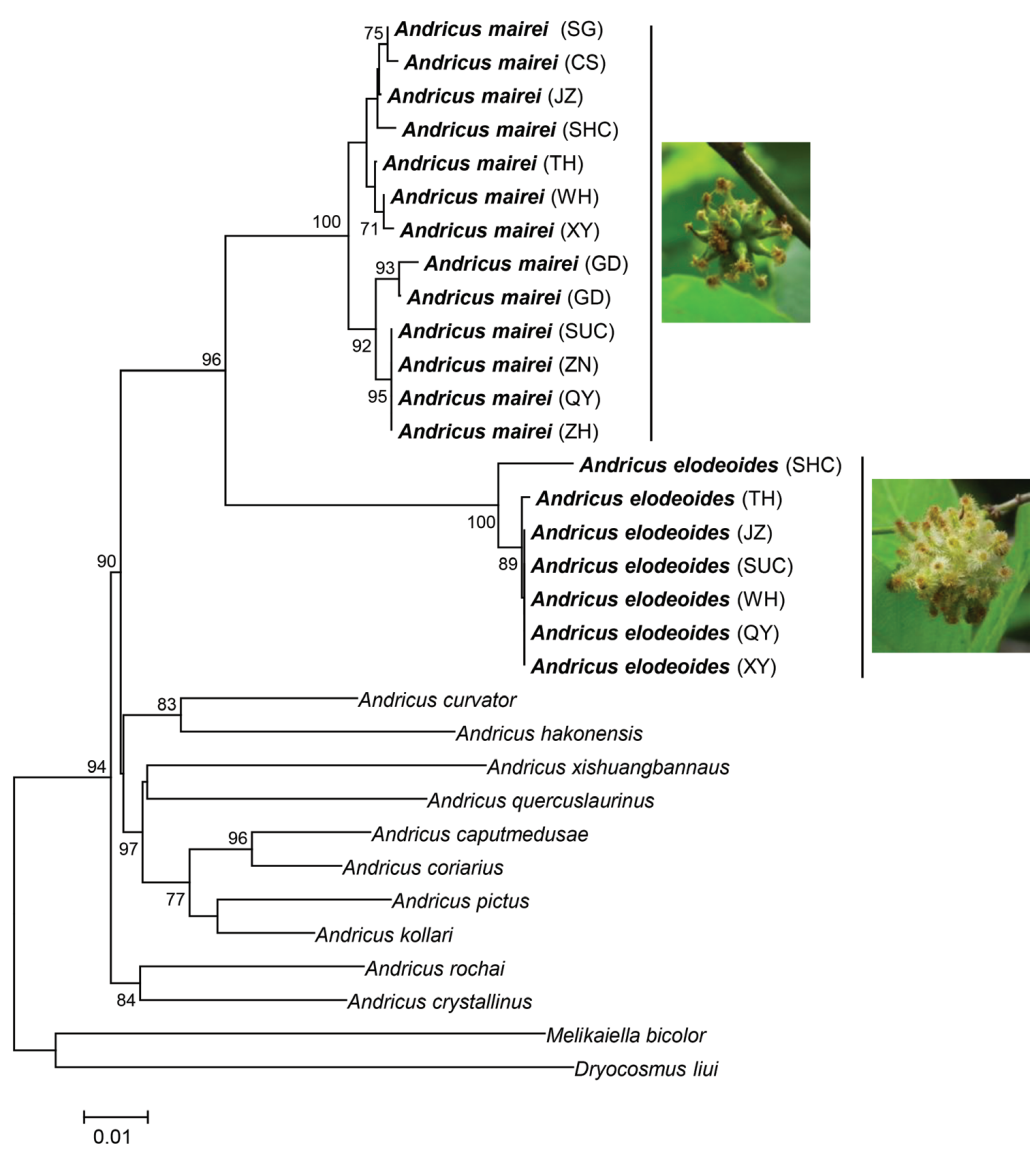
Bayesian phylogenetic tree of *A.elodeoides* sp. nov. and other *Andricus* species based on COI and 28S sequences. Bold font refers to the sequence obtained in this study, and others are downloaded from NCBI. The photograph on the right refers to the gall of adult emergence. The letters in parentheses indicate the sampled populations shown in Table 1. The length of the branches is drawn to scale of genetic distance and the number over branches is posterior probability. *Melikaiellabicolor* and *Dryocosmusliui* were used as the outgroup.

The Bayesian and maximum-likelihood phylogenetic trees of various populations of *A.elodeoides*, *A.mairei*, and other *Andricus* species based on the COI and 28S genes had identical topology while showing minor differences in support level for some nodes. According to the Bayesian trees presented here (Fig. 16), the sampled populations of *A.elodeoides* and *A.mairei* form their own monophyletic clades, and the two species are sister to each other. The genetic distance between the two species is similar to other *Andricus* species pairs, while the distance between this clade and the other including *Andricus* species is rather distinct (Fig. 16).

**Figure 17.**
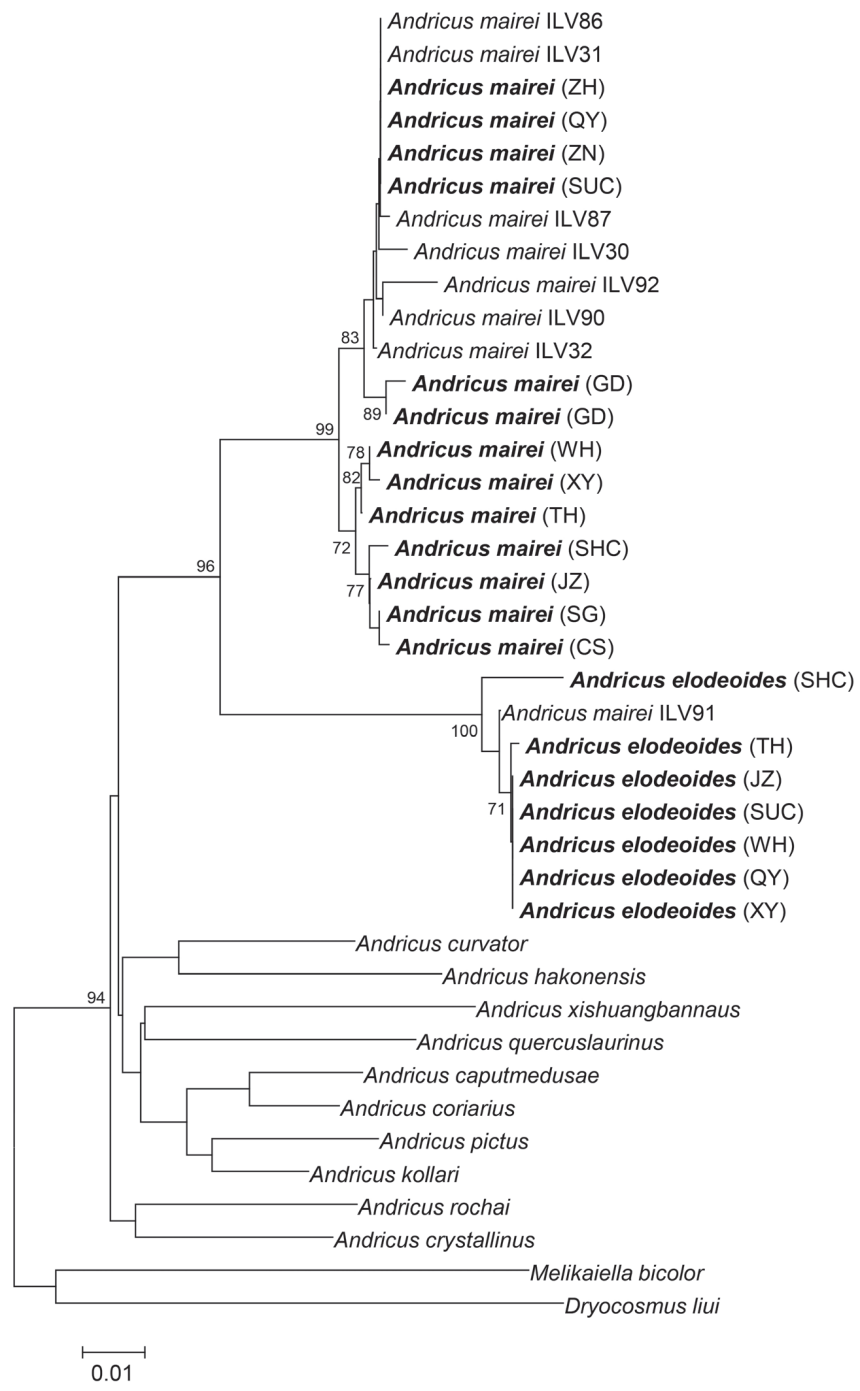
Bayesian phylogenetic tree for *A.elodeoides* sp. nov. and *A.mairei* of different geographic populations using COI sequences. Bold font refers to the sequence obtained in this study, and the others are from Pujade-Villar et al. (2020). The letters in parentheses indicate the sampled populations shown in Table 1. The length of the branches is drawn to scale and show the genetic distances, and the number over branches is posterior probability.

In the COI tree, all populations of *A.mairei* from Pujade-Villar et al. (2020) formed a single clade with our sampled populations of the species, except for “*A.mairei* ILV91” (MT179617) from Pujade-Villar et al. (2020), which fell into the *A.elodeoides* clade (Fig. 17).

Pair-wise comparison of the COI gene segment used in this study showed interspecific genetic distances ranged from 6.2 to 11.7% among *Andricus* species. In *A.elodeoides* and *A.mairei*, the interspecific genetic distance ranged from 6.2 to 8.9%. The level of intraspecific genetic variation in *A.mairei* was higher than that in *A.elodeoides*. The intraspecific genetic distances were 0–1.8% in *A.elodeoides* and 0–2.6% in *A.mairei*, while the distance between “*A.mairei* ILV91” and *A.elodeoides*, “*A.mairei* ILV91” and *A.mairei* were 0.2–1.8%, and 6.5–8.2%, respectively (Table 3).

**Table 3.** Pair-wise COI sequence distances in various geographic populations of *A.elodeoides* sp. nov. and *A.mairei*.

Species	1	2	3	4	5	6	7	8	9	10	11	12	13	14	15	16	17	18	19	20	21	22	23	24	25	26	27	28	29
1 *A.curvator*		0.010	0.011	0.011	0.011	0.011	0.011	0.011	0.011	0.011	0.011	0.011	0.011	0.011	0.011	0.014	0.011	0.011	0.011	0.011	0.011	0.011	0.012	0.012	0.011	0.011	0.011	0.011	0.011
2 *A.hakonensis*	0.070		0.013	0.013	0.012	0.013	0.012	0.012	0.012	0.013	0.012	0.012	0.012	0.013	0.012	0.015	0.012	0.012	0.012	0.012	0.012	0.012	0.014	0.014	0.013	0.013	0.013	0.013	0.013
3 *A.mairei* (SG*)	0.080	0.096																					0.010	0.010	0.010	0.010	0.010	0.010	0.010
4 *A.mairei* (CS)	0.082	0.096	0.002																				0.010	0.011	0.010	0.010	0.010	0.010	0.010
5 *A.mairei* (WH)	0.078	0.096	0.006	0.008																			0.010	0.010	0.010	0.010	0.010	0.010	0.010
6 *A.mairei* (TH)	0.080	0.098	0.005	0.006	0.002																		0.010	0.010	0.010	0.010	0.010	0.010	0.010
7 *A.mairei* (SUC)	0.082	0.092	0.014	0.015	0.011	0.009																	0.011	0.011	0.010	0.010	0.010	0.010	0.010
8 *A.mairei* (XY)	0.080	0.094	0.008	0.009	0.002	0.003	0.012																0.010	0.011	0.010	0.010	0.010	0.010	0.010
9 *A.mairei* (ZN)	0.082	0.092	0.014	0.015	0.011	0.009	0.000	0.012															0.011	0.011	0.010	0.010	0.010	0.010	0.010
10 *A.mairei* (JZ)	0.082	0.098	0.002	0.003	0.005	0.003	0.012	0.006	0.012														0.010	0.010	0.010	0.010	0.010	0.010	0.010
11 *A.mairei* (SHC)	0.082	0.094	0.005	0.006	0.008	0.006	0.015	0.009	0.015	0.003													0.010	0.010	0.010	0.010	0.010	0.010	0.010
12 *A.mairei* (QY)	0.082	0.092	0.014	0.015	0.011	0.009	0.000	0.012	0.000	0.012	0.015				*A.mairei*								0.011	0.011	0.010	0.010	0.010	0.010	0.010
13 *A.mairei* (GD1)	0.083	0.092	0.017	0.018	0.017	0.015	0.009	0.018	0.009	0.015	0.018	0.009											0.011	0.011	0.010	0.010	0.010	0.010	0.010
14 *A.mairei* (GD2)	0.083	0.096	0.014	0.015	0.014	0.012	0.006	0.015	0.006	0.012	0.015	0.006	0.003										0.011	0.011	0.010	0.010	0.010	0.010	0.010
15 *A.mairei* (ZH)	0.082	0.092	0.014	0.015	0.011	0.009	0.000	0.012	0.000	0.012	0.015	0.000	0.009	0.006									0.011	0.011	0.010	0.010	0.010	0.010	0.010
16 *A.mairei* ILV92^†^	0.091	0.099	0.022	0.024	0.024	0.024	0.011	0.026	0.011	0.020	0.022	0.011	0.020	0.015	0.011								0.013	0.013	0.012	0.012	0.013	0.012	0.012
17 *A.mairei* ILV90^†^	0.082	0.092	0.012	0.014	0.012	0.011	0.002	0.014	0.002	0.011	0.014	0.002	0.011	0.008	0.002	0.009							0.011	0.011	0.010	0.010	0.010	0.010	0.010
18 *A.mairei* ILV87	0.082	0.094	0.015	0.017	0.012	0.011	0.002	0.014	0.002	0.014	0.017	0.002	0.011	0.008	0.002	0.011	0.003						0.011	0.011	0.010	0.010	0.010	0.010	0.010
19 *A.mairei* ILV86^†^	0.081	0.092	0.014	0.015	0.011	0.009	0.000	0.012	0.000	0.012	0.015	0.000	0.009	0.006	0.000	0.011	0.002	0.002					0.011	0.011	0.010	0.010	0.010	0.010	
20 *A.mairei* ILV32^†^	0.081	0.092	0.012	0.014	0.012	0.011	0.002	0.014	0.002	0.011	0.014	0.002	0.008	0.005	0.002	0.009	0.003	0.003	0.002				0.011	0.011	0.010	0.010	0.010	0.010	
21 *A.mairei* ILV31^†^	0.081	0.092	0.014	0.015	0.011	0.009	0.000	0.012	0.000	0.012	0.015	0.000	0.009	0.006	0.000	0.011	0.002	0.002	0.000	0.002			0.011	0.011	0.010	0.010	0.010	0.010	0.0
22 *A.mairei* ILV30^†^	0.087	0.096	0.016	0.018	0.014	0.013	0.005	0.016	0.005	0.014	0.018	0.005	0.013	0.010	0.005	0.016	0.005	0.006	0.005	0.006	0.005		0.011	0.011	0.010	0.010	0.010	0.010	0.0
23 *A.elodeoides* (SHC)	0.097	0.117	0.076	0.078	0.078	0.080	0.083	0.080	0.083	0.078	0.078	0.083	0.085	0.085	0.083	0.089	0.082	0.086	0.084	0.084	0.084	0.084							
24 *A.mairei* ILV91^†^	0.090	0.103	0.066	0.068	0.066	0.066	0.070	0.068	0.070	0.068	0.068	0.070	0.072	0.072	0.070	0.082	0.072	0.070	0.070	0.070	0.070	0.071	0.018						
25 *A.elodeoides* (JZ)	0.087	0.105	0.070	0.071	0.068	0.070	0.073	0.070	0.073	0.071	0.071	0.073	0.075	0.075	0.073	0.079	0.076	0.076	0.074	0.074	0.074	0.075	0.017	0.002			*A.elodeoides*		
26 *A.elodeoides* (SUC)	0.087	0.105	0.070	0.071	0.068	0.070	0.073	0.070	0.073	0.071	0.071	0.073	0.075	0.075	0.073	0.079	0.076	0.076	0.074	0.074	0.074	0.075	0.017	0.002	0.000				
27 *A.elodeoides* (TH)	0.088	0.107	0.071	0.073	0.070	0.071	0.075	0.071	0.075	0.073	0.073	0.075	0.076	0.076	0.075	0.082	0.077	0.077	0.076	0.076	0.076	0.077	0.015	0.004	0.002	0.002			
28 *A.elodeoides* (WH)	0.087	0.105	0.070	0.071	0.068	0.070	0.073	0.070	0.073	0.071	0.071	0.073	0.075	0.075	0.073	0.079	0.076	0.076	0.074	0.074	0.074	0.075	0.017	0.002	0.000	0.000	0.002		
29 *A.elodeoides* (XL)	0.087	0.105	0.070	0.071	0.068	0.070	0.073	0.070	0.073	0.071	0.071	0.073	0.075	0.075	0.073	0.079	0.076	0.076	0.074	0.074	0.074	0.075	0.017	0.002	0.000	0.000	0.002	0.000	
30 *A.elodeoides* (XY)	0.087	0.105	0.070	0.071	0.068	0.070	0.073	0.070	0.073	0.071	0.071	0.073	0.075	0.075	0.073	0.079	0.076	0.076	0.074	0.074	0.074	0.075	0.017	0.002	0.000	0.000	0.002	0.000	0.000

*Indicates the population codes shown in Table 1 in this study, while ^†^ mean from Pujade-Villar et al. (2020).

### ﻿*Wolbachia* infection

Using PCR screening for *Wolbachia* infection with *wsp* gene-specific primers, in all sampled populations of *A.elodeoides* and *A.mairei*, we found that all individuals from 12 populations of *A.mairei* (*N* = 360) were infected with *Wolbachia*, whereas no *Wolbachia* infection was found in the seven studied populations of *A.elodeoides* (*N* = 350), including samples collected from Jinzhai and Shucheng populations through four consecutive years (Table 1). The negative results of *Wolbachia* infection in *A.elodeoides* adults were further verified by PCR using specific primers for the multilocus sequence type genes (*ftsZ*, *coxA*, *hcpA*, and *gatB*).

## ﻿Discussion

*Andricuselodeoides* sp. nov. is considered a distinct from *A.mairei* (Kieffer) based on differences in adult and gall morphology, and phylogenetic reconstruction based on COI sequence data (Fig. 17), as well as combined dataset of 28S and COI genes (Fig. 16) and pair-wise genetic distance of the COI gene marker (Table 3). However, intraspecific variation of adult morphology exists in *A.elodeoides* as well as in *A.mairei* (Pujade-Villar et al. 2020). For example, the median propodeal area is rugose in specimens from Hunan (Changsha and Yueyang), but smooth in specimens from Guizhou (Guiding) and Fujian. The lateral propodeal carinae are parallel to each other in *A.elodeoides*, as we observed, which appear to be highly variable in *A.mairei* from being “subparallel to divergent anteriorly and bent outwards in the middle” (Pujade-Villar et al. 2020). Such variations in the morphology of both species, while needing to be further evaluated using large series of specimens from broad regional populations, certainly make it difficult to separate the two species based on adult morphology alone. In such situations, gall morphology and DNA barcoding based on COI sequence is necessary.

Pujade-Villar et al. (2020) suspected that one of specimens included in their study as *A.mairei* (ILV91) was probably a new species based on the COI genetic distance. Our COI tree including this sequence (Fig. 17) and our pairwise genetic distance analysis (Table 3) supported their hypothesis. In addition, galls in one photograph in that paper (Pujade-Villar et al. 2020: fig. 7b) very likely belonged to *A.elodeoides*, although it is not clear to us whether these galls were the same as those which *A.mairei*-ILV91 was reared from.

Our phylogenetic analyses of gene sequence data support *A.elodeoides* and *A.mairei* as sister species (Figs 16, 17). The two species are sympatric in distribution and share the same host plant species, make galls on the same host plant structure (the stalk of male catkins), and overlap in time of gall formation and the emergence of adults. In addition, the galls of the two species share striking structural similarities despite distinct morphological differences (Figs 14, 15). Given these facts, it is intriguing what speciation mechanisms might have been involved given the lack of barriers in biogeography, host plant use, and phenology between the two species. It is possible that *Wolbachia*-induced cytoplasmic incompatibility was one of the potential causes for speciation between *A.mairei*, which is infected with *Wolbachia*, and its uninfected sister species *A.elodeoides*. Nonetheless, we did not conduct interspecific mating experiments after curing of *Wolbachia* due to the difficulties in artificial breeding of gall wasps.

*Wolbachia* (Anaplasmataceae) are maternally inherited endosymbiotic bacteria that infect arthropods and nematodes and has been shown to be associated with multiple effects on the reproduction of their hosts, such as cytoplasmic incompatibility (CI), induced parthenogenesis, feminization of genetic males, and male killing (Werren et al. 2008). Several studies have revealed *Wolbachia* infection in diverse cynipid species, involving tribe Aylacini, Diplolepidini, Cynipini, and Synergini (Plantard et al. 1998; Abe and Miura 2002; Zhu et al. 2007; Yang et al. 2013; Hou et al. 2020). In this study, we found that all examined individuals of *A.mairei* were infected with *Wolbachia*, whereas individuals of *A.elodeoides* collected from seven sites were all *Wolbachia*-free. Reproductive isolation between different populations or incipient species can evolve in both sympatry and allopatry (Turelli and Bierzychudek 2001). In arthropods, sympatry isolation may result from infection by *Wolbachia* reproductive manipulators (Engelstädter and Hurst 2009; Weinert et al. 2015). Cytoplasmic incompatibility, the most common form of reproductive manipulation induced by *Wolbachia* to its hosts, is characterized by partial or complete embryonic lethality in crosses between infected males and uninfected females or between hosts carrying incompatible symbiont strains. Thus, *Wolbachia*-induced CI may create substantial barriers to genetic exchange between individuals with different infection status and act as an agent of speciation (Werren 1998; Wade 2001; Turelli 2010). Bordenstein et al. (2001) reported a preeminent case of symbiont-assisted isolation because of *Wolbachia*-induced CI in the parasitoid wasp genus *Nasonia* (Hymenoptera, Chalcidoidea). This study demonstrated that *Wolbachia*-induced reproductive isolation via CI preceded the evolution of other mating barriers in *Nasonia* species and was the first major step in the process of speciation.

A contrasting difference in sex ratio was observed between *A.elodeoides* and *A.mairei*. Populations of of *A.elodeoides* were extremely female-biased, with female rates being 95.5–97.8%, while populations of *A.mairei* were more male biased to nearly balanced, with female rates being 5.4–43.5%. For two *A.mairei* populations in Jinzhai and Shucheng, which were investigated for six and four consecutive years, the female rates were 17.3% and 24.6%, or lower, respectively. This is consistent with observations made by other studies. Weld (1952) reported that there was only one female among the six adults of *A.mairei* collected from Hankou. Yang et al. (2012) collected specimens from multiple locations, including Yueyang, Changsha and Shaoyang, in Hunan Province, with a female ratio of less than 20%. The contrasting sex ratio biases of *A.elodeoides* and *A.mairei* are an interesting phenomenon that might be associated with *Wolbachia* infection. Genetic mutation or recombination may result in differences in susceptibility to *Wolbachia* infection in gall wasps and somehow effectively interrupted the genetic exchange between genotypes by mechanisms mentioned above. Consequently, a sympatric speciation event could take place relatively quickly due to founder effect (Joly 2011). This may explain our observation that the COI genetic distance between *A.elodeoides* and *A.mairei* is comparable to the average distance among known *Andricus* species from Eastern Asian while the two species are very similar in morphology, phenology, and gall morphology (Table 3). Nonetheless, the exact mechanism involved could only be understood by further investigations.

## Supplementary Material

XML Treatment for Andricus elodeoides
